# Synuclein Family Members Prevent Membrane Damage by Counteracting α-Synuclein Aggregation

**DOI:** 10.3390/biom11081067

**Published:** 2021-07-21

**Authors:** Christian Scheibe, Christiaan Karreman, Stefan Schildknecht, Marcel Leist, Karin Hauser

**Affiliations:** 1 Department of Chemistry, University of Konstanz, 78457 Konstanz, Germany; christian.scheibe@uni-konstanz.de; 2 Department of Biology, University of Konstanz, 78457 Konstanz, Germany; christiaan.karreman@uni-konstanz.de (C.K.); schildknecht@hs-albsig.de (S.S.); marcel.leist@uni-konstanz.de (M.L.); 3 Department of Life Sciences, Albstadt-Sigmaringen University of Applied Sciences, 72488 Sigmaringen, Germany

**Keywords:** polarized ATR-FTIR, protein–membrane interaction, protein–protein interaction, alpha-synuclein, beta-synuclein

## Abstract

The 140 amino acid protein α-synuclein (αS) is an intrinsically disordered protein (IDP) with various roles and locations in healthy neurons that plays a key role in Parkinson’s disease (PD). Contact with biomembranes can lead to α-helical conformations, but can also act as s seeding event for aggregation and a predominant β-sheet conformation. In PD patients, αS is found to aggregate in various fibrillary structures, and the shift in aggregation and localization is associated with disease progression. Besides full-length αS, several related polypeptides are present in neurons. The role of many αS-related proteins in the aggregation of αS itself is not fully understood Two of these potential aggregation modifiers are the αS splicing variant αS Δexon3 (Δ3) and the paralog β-synuclein (βS). Here, polarized ATR-FTIR spectroscopy was used to study the membrane interaction of these proteins individually and in various combinations. The method allowed a continuous monitoring of both the lipid structure of biomimetic membranes and the aggregation state of αS and related proteins. The use of polarized light also revealed the orientation of secondary structure elements. While αS led to a destruction of the lipid membrane upon membrane-catalyzed aggregation, βS and Δ3 aggregated significantly less, and they did not harm the membrane. Moreover, the latter proteins reduced the membrane damage triggered by αS. There were no major differences in the membrane interaction for the different synuclein variants. In combination, these observations suggest that the formation of particular protein aggregates is the major driving force for αS-driven membrane damage. The misbalance of αS, βS, and Δ3 might therefore play a crucial role in neurodegenerative disease.

## 1. Introduction

Neurodegenerative diseases are becoming more and more prevalent as the average age of the population increases [1]. Parkinson’s disease (PD) is linked to α-synuclein (αS) [2], a protein that belongs to the intrinsically disordered proteins (IDPs). It consists of 140 amino acids (aa) and is disordered in solution. Previous studies suggest that at least part of its physiological function is linked to the organization of presynaptic vesicles [3,4,5,6,7]. It has been shown that aggregation of αS into β-sheet rich fibrillary structures causes deleterious effects on the cell membrane. Furthermore, changes in protein aggregation have been suggested to play a role in the pathogenesis of PD [8,9,10,11]. Oligomeric αS intermediates are hypothesized to permeabilize cellular membranes, and potential molecular mechanisms of membrane disruption have been discussed [12,13,14]. Aggregation of αS increases with the concentration of αS [15]. The N-terminal part of αS binds to negatively charged lipid membranes and forms α-helices [16,17,18,19,20]. It has also been shown that binding to a membrane enhances the aggregation process [21]. In the membrane bound state, the C-terminal part of αS remains in solution and is still disordered. Several studies have shown that different metal ions can bind to the C-terminus [22,23,24,25], and it has also been reported that the C-terminus can interact with zwitterionic lipids [26,27]. Disease mutants found in familial forms of PD have received a lot of attention. Several point mutations leading to early (A30P, E46K, G51D, A53T) or late (H50Q) disease onset have been identified [28,29,30,31,32,33]. Furthermore, it has been shown that different point mutations are able to alter the interaction of the protein with phospholipid membranes [34].

Although many studies have already been carried out investigating αS, its physiological role and its role in PD, there is still a lot to be elucidated. In particular, the impact of the other members of the synuclein family, β-synuclein (βS) and γ-synuclein (γS), as well as the splicing variant αS Δexon3 (Δ3), which are all present in the brain, have not been studied much so far [35,36]. βS consists of 134 aa and the N-terminus has great homology to the N-terminus of αS. However, βS lacks 11 aa within the central NAC region in comparison to αS. Like αS, βS has been shown to interact with lipid vesicles and to adopt an α-helical structure upon binding to lipid membranes [37]. Both proteins are present in the presynaptic terminals and expressed at similar levels [38,39]. αS is reported to physiologically occur in low micromolar concentrations [40]. Due to their high level of sequence similarity, it has been suggested that αS, βS and γS could carry out redundant physiological roles. This is supported by triple-knockout mice having more severe phenotypes compared to single and double αS, βS, and γS knockout models [41]. Unlike αS, βS does not aggregate in fibrillar structures under physiological conditions [42]. Fibrillation of βS can be induced by acidic pH [43], metal ions, and certain pesticides [44] or at elevated temperatures in the presence of lipid vesicles [45]. However, the E61A mutation in βS removes the pH dependence of βS fibril formation and leads to a fibril formation at a comparable rate to αS at neutral pH [43]. Furthermore, the missense mutations V70M and P123H of βS have been linked to dementia with Lewy bodies (DLB) [46]. Altered expression levels of αS and βS have been reported to have an impact on the onset of PD [47]. βS has been described as having a neuroprotective function by inhibiting the aggregation of αS [48,49,50,51,52,53] and especially inhibiting the so-called secondary nucleation pathway, describing the aggregation of αS at the surface of pre-existing amyloid fibrils [49]. This is assumed to be due to the formation of hetero-dimers and competitive binding to surfaces [54]. βS inhibits the aggregation of wild type αS, and the mutants A30P and G51D, but not the aggregation of E46K, H50Q, or A53T [54]. Overexpression of βS has been reported to be neurotoxic, but to a lesser extent than the overexpression of αS [55].

Δ3 is a physiologically occurring alternative splicing variant of αS that lacks the 3rd exon consisting of 14 amino acids from positions 41 to 54. This exon is especially interesting since it contains four of the five most common disease mutants [56] and it is part of the N-terminus interacting with lipid membranes. Since the N-terminal part of this variant is shorter, with the C-terminus being fully intact, this leads to a more negative net charge of the protein compared to the full length αS. This is expected to decrease its aggregation properties and confer anti-aggregation properties to this variant [57]. However, it has also been shown that Δ3 is able to form fibrils in vitro [58]. Furthermore, Δ3 shows a reduced ability to bind to plasma membranes due to the interrupted membrane binding domain [59]. Δ3 displays no toxicity on its own but has been reported to induce αS toxicity [59]. Δ3 is expressed at low levels compared to αS [35]. Patients with DLB and AD show diminished levels of the corresponding mRNA in the frontal cortex, while patients with PD have an increased expression in the same area [60]. For patients with diminished Δ3 levels, Lewy pathology has been observed, but not for patients with increased levels of Δ3 [57]. Since synuclein variants are present in the brains of all PD patients, a deeper insight into the interplay between the variants is desired. It is reported that only about 13% of PD patients show a family history of the disease caused by disease mutations [61], thus studying intermolecular interactions of synuclein variants will be beneficial for a mechanistic understanding of PD.

Attenuated total reflection Fourier-transform infrared (ATR-FTIR) is a highly sensitive method of monitoring protein–membrane interactions [62]. By use of a solid supported lipid bilayer (SSLB) as a biomimetic membrane, synuclein aggregation and membrane integrity can be observed simultaneously as we have shown before [21]. ATR-FTIR spectroscopy with polarized light provides insights into molecular orientations and we previously analyzed orientation changes of lipid molecules within a membrane [63]. In this study, we investigate the impact of βS and Δ3 on membrane-induced aggregation of αS by ATR-FTIR spectroscopy. The focus is set on the interrelation between synuclein variants. It is analyzed whether the presence of another synuclein alters membrane interaction and aggregation of αS. Experiments are performed with synuclein mixtures at different ratios. Our methodological approach allows us to study the membrane itself, the secondary structure of the proteins, and the orientation of molecular groups all at the same time. Membrane damage is quantified by changes in lipid surface concentrations upon protein interaction. Our measurements elucidate whether βS can prevent αS aggregation and membrane damage and if Δ3 could fulfill a similar neuroprotective function as is suspected for βS.

## 2. Materials and Methods

### 2.1. Protein Expression and Purification

The coding segments for αS and βS genes were obtained by PCR on cDNA complementary to mRNA of synuclein expressing cells. Synuclein expressing cells were generated from the ES cell line H9 using a protocol developed by S. Klima [64]. The splice variant of αS missing the third exon was generated from full length αS using a variant of the fusion PCR method [65].

Genes were recloned into the pET11c vector [66] and transfected into the *E*. *coli* strain Tuner (DE3). Cells were grown in a volume of 1 L to an OD of 0.6 at 37 °C and the expression of the proteins was started by the addition of IPTG (final concentration of 1 mM). Cells were grown for another two hours and subsequently harvested by centrifugation. The pellets were resuspended in 25 mL of PBS and the cells were lysed by putting the suspension in boiling water for two minutes. The lysis was cleared by centrifugation and the supernatant was further used. The proteins were isolated in one step from the crude mixture by chromatography using capture select beads (Thermo Fisher Scientific, Darmstadt, Germany). The resulting peak fractions were desalted and lyophilized. Dry pellets were kept at −20 °C until needed, upon which they were dissolved in water, aliquoted in ready to use quantities and kept in liquid nitrogen until the start of the measurements. Further details can be found in the Supporting Information (SI).

### 2.2. Biomimetic Membranes 

Solid supported lipid bilayers (SSLBs) were prepared by spreading small unilamellar vesicles (SUVs) on the internal reflection element (IRE) of the ATR-cell. SUVs were produced using 1-palmitoyl-2-sn-glycero-3-phosphocholine (POPC) and 1-palmitoyl-2-sn-glycero-3-phospho-rac-(1′-glycerol) (POPG) from Avanti Polar Lipids, Alabaster, AL, USA with a 1:1 ratio. To get SUVs, POPC and POPG were dissolved in chloroform at 25 mg/mL. A total of 100 µL of each lipid solution were mixed in a small glass bottle, dried under a gentle nitrogen stream for 5 min, and put in a vacuum chamber for two hours. Afterwards, the lipids were resuspended in 1 mL Tris-HCl (10 mM, 5 mM MgCl, pH 7.4). After 30 min of incubation at room temperature, the lipid solution was treated with a tip sonicator (Hielscher Ultrasound Technology, Teltow, Germany). This step was repeated four times at 15 min intervals. The sonication was followed by extrusion using a handheld extruder from Avanti Polar Lipids with a 30 nm polycarbonate membrane.

In preparation for the formation of an SSLB, the IRE was polished using a smooth cloth and polishing paste. The IRE was treated with H_2_SO_4_ (95%) for 10 min, rinsed with water, and dried under a gentle nitrogen stream. This procedure was repeated 3 times to achieve a more polar surface and to enhance the spreading of the SUVs. After adding the SUVs to the IRE, the formation of the bilayer was monitored spectroscopically for 40 min at 25 °C. Afterwards, the IRE was rinsed using water to remove leftover vesicles from the supernatant. Buffer was added and a spectrum was recorded to verify if full coverage of the IRE with an SSLB has been achieved.

### 2.3. Sample Preparation

After expression, the proteins were dissolved in MQ-water at 1 mg/mL, 60 µL were put into a small tube, frozen, and stored in liquid nitrogen to avoid aggregation until being used in an experiment. The protein samples were thawed immediately before adding them to the prepared SSLB. This procedure was done to prevent premature aggregation as much as possible. For experiments with a single synuclein variant, 50 µL of freshly thawed protein solution were added. For experiments with protein mixtures, the two variants were mixed at a defined ratio.

### 2.4. Polarized ATR-FTIR

FTIR spectra were recorded using a Vertex 70v spectrometer (Bruker Optics, Germany) and a Bio-ATR II cell (Bruker Optics, Ettlingen, Germany). Additionally, an infrared polarizer (PIKE Technologies, USA) was used to enable measurements with polarized light to determine the orientation of molecular groups. The alignment of the polarizer was remotely controlled. The IRE consists of silicon with a refractive index of n_1_ = 3.42. An angle of incidence of 45° and a refractive index of the sample of n_2_ = 1.55 results in a penetration depth of 0.42 µm for perpendicular and 0.84 µm for parallel polarized light at 1650 cm^−1^ [67]. More details about the evanescent field and the calculation of the penetration depth are given in SI. For each spectrum, 32 scans were recorded with a resolution of 4 cm^−1^. The sample chamber was flushed with dry air during the experiments and the temperature of the ATR cell was set to 25 °C using a water bath with an integrated E300 immersion thermostat (LAUDA-Brinkmann, Delran, NJ, USA).

Prior to the experiments, background spectra were recorded. For the analysis of the surface concentration of lipids, the background spectra contain only the buffer solution on the IRE. For the analysis of the protein variants, background spectra were recorded after the formation of the SSLB.

The surface concentration of the lipids was calculated using Γ = (A_‖_n_12_cosγ)/(Nε(E_x_^2^ + E_z_^2^)) = (A_⊥_n_12_cosγ)/(NεE_y_^2^) [68]. A_‖_ and A_⊥_ are the integrated absorbances of the corresponding bands of the lipid alkyl chains at 2854 cm^−1^ and 2924 cm^−1^ for parallel or perpendicular polarized light, n_12_ is the refractive index of the IRE divided by the refractive index of the sample (lipids: n_2_ = 1.43 [68], proteins: n_2_ = 1.45 [69]), γ is the incidence angle, N is the number of active internal reflections (9–12, for calculations N = 10.5 has been used), ε is the molecular extinction coefficient for lipids and E_xyz_ are the relative electric field components of the evanescent field in the x-, y- and z-direction [68] (E_x_ = 1.3916, E_y_ = 1.5350, E_z_ = 1.2268). The surface concentration was calculated after every preparation of an SSLB to ensure that the IRE was completely covered. Other studies reported a surface concentration of 230 pmol/cm^2^ for a POPC monolayer [68] and 448 ± 57 pmol/cm^2^ for a POPC bilayer [70]. Since POPC and POPG only differ in the headgroup, it has been assumed that the surface concentration for a POPC:POPG bilayer is the same as for a bilayer consisting of only POPC. At a surface concentration of 460 pmol/cm^2^, complete coverage of the IRE with an SSLB was assumed.

To investigate the orientation of secondary structure elements, dichroic spectra were used. Dichroic spectra were calculated by subtracting s-polarized spectra from p-polarized spectra applying self-written scripts in MatLab (MathWorks, Natick, MA, USA). Negative values in the amide I region of the dichroic spectra indicate a structure being oriented perpendicular to the surface normal of the IRE while positive values correspond to a structure being oriented parallel to the surface normal [67]. More details on the analysis of polarized ATR-FTIR measurements are given in SI.

Most of the detected IR signals were very small, thus at least four independent experiments were conducted for each sample to ensure reproducibility. The shown spectra and the values for the surface concentrations are averages of the repeated experiments.

## 3. Results & Discussion

**Figure 1:**
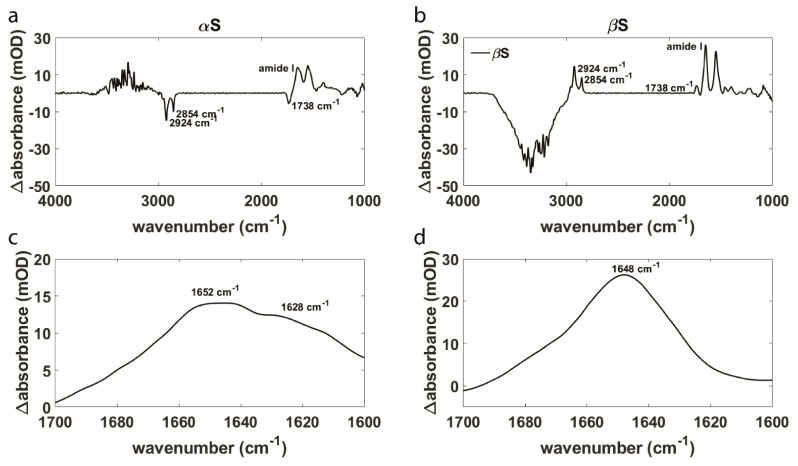
(**a**,**b**) Difference spectra of αS and βS after 1 h of interaction with an SSLB (**c**,**d**) Magnification of the amide I band showing differences in the secondary structure of αS and βS.

The conformation of a protein is indicated by the shape of the amide I band, a broad band between 1600 cm^−1^ and 1700 cm^−1^ which is composed of several amide I components corresponding mainly to the carbonyl stretching vibrations of the protein backbone. The absorption maxima of the amide I components at different wavenumbers give information about the composition of secondary structure elements of the protein. We investigated the effect of protein–membrane interaction on the conformation of αS, βS, and Δ3 as well as the effect on the integrity of the membrane. In solution, αS is disordered and the amide I band maximum occurs between 1640 cm^−1^ and 1644 cm^−1^. When the protein adopts an α-helical conformation upon interaction with an SSLB, the maximum of the absorption shifts to wavenumbers higher than 1650 cm^−1^ [21]. In the aggregated form αS is rich in β-sheets that absorb, depending on their extension, between 1618 cm^−1^ and 1630 cm^−1^ [71,72]. Additionally, the spectra show bands that can be attributed to the lipids within the membrane as well as bands resulting from water absorption. The carbonyl stretching vibration of the lipids is around 1738 cm^−1^ and the symmetric and antisymmetric stretching vibrations of the alkyl groups are between 2800 cm^−1^ and 3000 cm^−1^ [68]. The O-H stretching vibration of water absorbs between 3000 cm^−1^ and 3700 cm^−1^ and the O-H bending vibration around 1645 cm^−1^ [67]. The latter has an impact on the amide I absorption, but it is still possible to detect changes within the secondary structure of the protein. Figure 1a,b show the spectra of αS and βS after 1 h of interaction with an SSLB. The sign of the lipid and water bands contain information about the integrity of the lipid membrane. Negative lipid bands in combination with positive water bands indicate that lipid molecules are displaced from the SSLB and at the same time water molecules are getting closer to the IRE surface [21,63]. Lipid molecules are no longer part of the SSLB and water molecules can only get closer to the crystal if the SSLB covers less of the surface of the IRE. Therefore, this is interpreted as a disruption of the membrane. It is possible that individual soluble lipid molecules are involved in a membrane insertion process [14], but we could not observe such an interaction with our experimental approach as further discussed below.

### 3.1. Aggregation Behavior and Membrane Interaction Are Significantly Different for α- and β-Synuclein

For the investigation of the interaction of synuclein variants with phospholipid bilayers, ATR-FTIR measurements were performed. Figure 1 shows difference spectra of αS and βS 1 h after the corresponding variant has been added to the membrane. The background spectra recorded prior to the experiments contain the SSLB and buffer solution. The secondary structure and the conformational changes of the proteins were analyzed. Upon interaction with lipid membranes, the spectra of αS and βS show significant differences. While the amide I band of αS has a distinct band component at 1628 cm^−1^ (Figure 1c) such a component is not visible for βS (Figure 1d). Furthermore, both amide bands have their maximum around 1650 cm^−1^. This indicates that both variants adopt a partially α-helical structure when in contact with a lipid membrane. Following this, αS shows an increase in absorption at around 1628 cm^−1^, which corresponds to an increase in β-sheet content. These findings are consistent with our previous studies on αS-membrane interaction where we analyzed time-dependent measurements revealing an initial α-helical structure upon membrane binding and subsequent occurrence of β-structured aggregates [21]. Here, we do not observe the formation of β-structure for βS. This indicates that the initial interaction of αS and βS with the lipid membrane is similar. Differences occur over time because αS aggregates while βS remains in its partially α-helical conformation.

Furthermore, changes in the lipid membrane are observed. It is striking that for αS all bands attributed to the lipids are negative while the water band is positive. For βS, we observe the opposite. As the strength of the evanescent field decays exponentially with the distance to the surface of the IRE, negative bands indicate reduced absorption, which can often be attributed to molecules getting further away from the crystal surface. Positive bands, on the other hand, can be interpreted as molecules getting closer to the IRE. This leads to the conclusion that upon interaction of αS with an SSLB, lipid molecules are removed from the IRE surface. The latter is interpreted as membrane damage. In contrast, the lack of absorption at 1628 cm^−1^ and the fact that there are no negative lipid bands in the spectra for βS indicate that there is neither aggregation nor perturbation of the lipid membrane. This is in line with previous studies that assume that aggregation of αS is causing deleterious effects on the cell membrane in Parkinson’s disease [8,9,10] and with studies that propose a protective effect of βS [48,49,50,51,52,53].

### 3.2. Synuclein Interaction Prevents α-Synuclein Aggregation and Maintains Membrane Integrity

To investigate further the perturbation of the membrane upon synuclein interaction and to get a quantitative estimate of the degree of damage, the surface concentration of the lipids on the IRE was determined over time as described in Methods and Materials. Different samples were used, and experiments were repeated several times. The surface concentration is calculated relative to the initial surface concentration before interaction with the protein. 

For validation of the feasibility of this approach to quantify membrane damage, control experiments were conducted. The membrane was purposely destroyed by using sodium dodecyl sulfate (SDS). In addition to this, poly-L-lysine (PLL) was added to the membrane since it is expected to bind to the membrane without causing damage [21]. We expect to no longer have an intact SSLB covering the IRE completely when the membrane is perturbed. If there are areas of the IRE that are no longer covered by lipids, water molecules can get closer to the surface of the IRE. This would result in positive water signals. The opposite is expected for the lipid signal. Less coverage of the SSLB means that lipid molecules are removed from the surface. This kind of experiment has been done in the past to validate that negative lipid bands indicate membrane damage [21]. Here, we determined additionally the lipid surface concentration as indicator for membrane damage.

**Figure 2:**
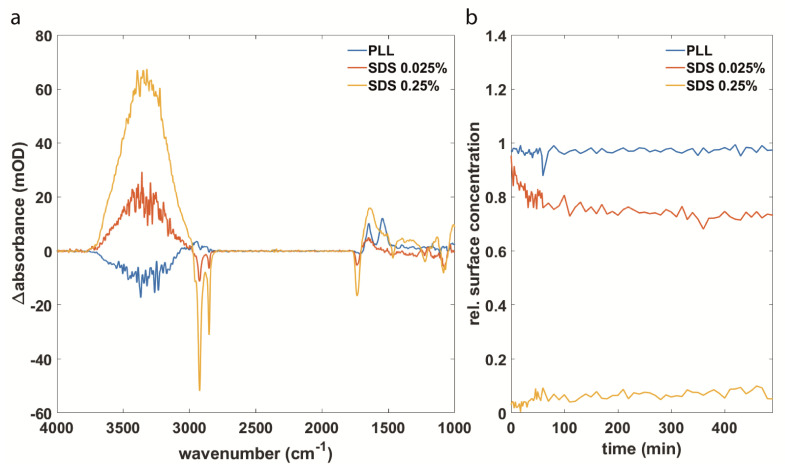
(**a**) Difference spectra of PLL, 0.025% SDS and 0.25% SDS after 1 h of interaction with an SSLB. (**b**) The surface concentration of lipids over time upon interaction with the same samples as in (**a**).

The results for the control experiments are shown in Figure 2. The difference spectra of SDS interacting with the SSLB (Figure 2a) reveal negative lipid bands and a strong positive water signal between 3700 cm^−1^ and 3000 cm^−1^. The signs of these bands are equal to those observed for the membrane interaction with αS. The difference spectra for PLL show no negative lipid bands and a small negative water band due to the displacement of water from the surface as PLL binds to the membrane [73]. This is in line with the determination of the relative surface concentrations. The relative surface concentration plotted in Figure 2b is calculated by dividing the surface concentration after adding SDS or PLL to the surface concentration before adding the sample. By plotting the relative surface concentration, it is easier to compare experiments with slightly different starting concentrations and it directly shows the fraction of the damaged membrane. A relative surface concentration of 1 corresponds to no change in the surface concentration, while values < 1 indicate a decrease in surface concentration and values > 1 an increase in surface concentration. For PLL, no change over 8 h is observed as expected [21]. For the two different SDS concentrations, we see a decrease in lipid surface concentration. In the experiments using 0.25% of SDS, the surface concentration of the lipids is immediately near zero and stays constant over the whole experiment. This is interpreted as immediate removal of the whole membrane from the IRE surface and the process is faster than the time-resolution of our experiment. For 0.025% of SDS, a decrease over time is observed until the surface concentration stabilizes at roughly 70% of the initial concentration. From these results, we conclude that this method is suitable to quantify membrane damage and to compare the effect of different synuclein variants on phospholipid membranes.

**Figure 3:**
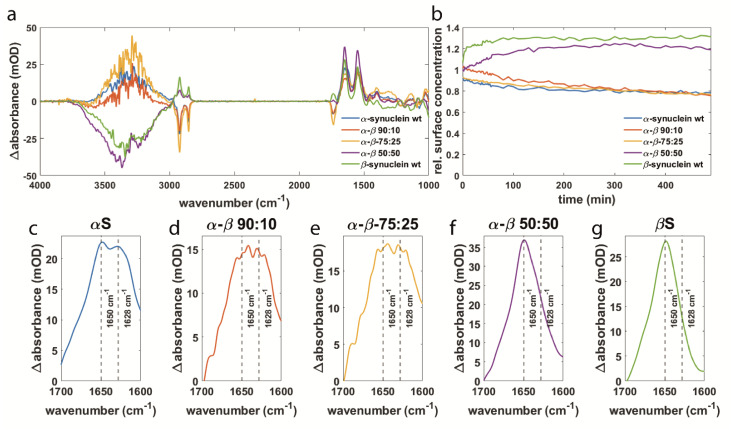
(**a**) Difference spectra of αS and βS and mixtures of the proteins at ratios of 90:10, 75:25, and 50:50 after 8 h of interaction with an SSLB. (**b**) Relative surface concentration of the lipids upon interaction with the samples from (**a**). (**c**–**g**) Magnification of the amide I band from the spectra in (**a**) with decreasing αS and increasing βS amounts.

The effects of αS and βS on the membrane and how these variants influence each other’s interaction were determined by the lipid surface concentration for both variants as well as for the synuclein mixtures at different ratios. Figure 3a shows the spectra after 8 h of interaction. The spectrum for αS reveals negative lipid bands at 2854 cm^−1^ and 2924 cm^−1^ and a positive water signal at 3000–3800 cm^−1^. The spectrum for βS contains positive lipid bands and a negative water signal. For the 50:50 mixture of both variants, we observe a spectrum that compares rather to the spectrum of βS than to αS. The signs of the lipid bands and the water signal are the same as in the βS spectrum and opposite compared to the αS spectrum. With increasing ratio of αS to βS, the resulting spectra compare more to the ones for pure αS. This is also reflected in the surface concentrations of the lipids as seen in Figure 3b. For αS, a decrease in the surface concentration is observed like the one for 0.025% of SDS. These data indicate a perturbation of the membrane but not complete removal since this would result in spectra comparable to those for 0.25% of SDS. For βS and the 50:50 mixture of both variants, even an increase in the surface concentration is observed. This implies that not only the damage on the membrane is prevented but also that more lipid molecules get closer to the IRE. Since both variants are expressed and purified in the same way and all lipids are expected to be removed during the purification process, it is not assumed that additional lipids are added with the βS sample. Previously, it has been described that the C-terminus of αS is able to interact with zwitterionic lipids like POPC [26,27]. Binding lipid molecules might be part of its physiological function in the organization of presynaptic vesicles [3,4,5,6,7]. Considering the here observed increase in lipid surface concentration for a 50:50 mixture, it seems that preventing αS aggregation allows the free C-termini to bind lipids from the supernatant. When there are no free C-termini due to αS aggregation, the binding of lipid molecules in proximity to the membrane is lost. An increase in lipid concentration is also observed for pure βS, which also binds to the membrane without aggregation so that the free C-termini can interact with lipids from the supernatant.

The data with a significant excess of αS (α-β 90:10 and α-β 75:25) reveal that the prevention of membrane damage is dependent on the fraction of βS in the mixture relative to αS. It seems that βS can only affect the membrane interaction of αS if both variants are present in comparable proportions. With an excess of αS, no prevention of membrane damage is observed meaning that βS needs to occur in equal or higher concentrations compared to αS to fulfill a membrane protecting function.

In Figure 3c–g it becomes obvious that the amide I component at ~1628 cm^−1^, which indicates aggregate formation, is reduced the more βS is present in the mixture. This suggests that the decrease in membrane damage as seen in Figure 3b is linked to a decrease in aggregation of αS. Our results support other studies using CD spectroscopy, ThT fluorescence, and atomic force microscopy that also reported less aggregation of αS upon membrane binding in the presence of βS as well as an inhibition of the secondary nucleation pathway [49]. It shows that the effect of aggregation on membrane integrity is highly dependent on the concentration ratio of αS and βS and that a similar amount of βS compared to αS needs to be present to impact αS aggregation. In summary, we showed that interaction with βS hinders αS aggregation and prevents membrane damage. A low concentration of βS in the presynaptic terminals could be one of the factors contributing to PD.

### 3.3. αS Δexon3 Shows Similar Effects to β-Synuclein but to a Lesser Extent

As for βS, the physiological role of Δ3 has not yet been clearly resolved. Both variants are always present in human neurons [35,36]. It has been suggested that Δ3 could be an aggregation preventing isoform due to the interruption of the membrane–protein interacting domain and its decreased expression levels in dementia with Lewy bodies (DLB), Lewy body variant of Alzheimer disease (LBVAD), and Alzheimer Disease (AD) [74]. For a better understanding of the molecular interaction mechanisms and function of Δ3, the same mixing experiments have been performed as for βS.

**Figure 4:**
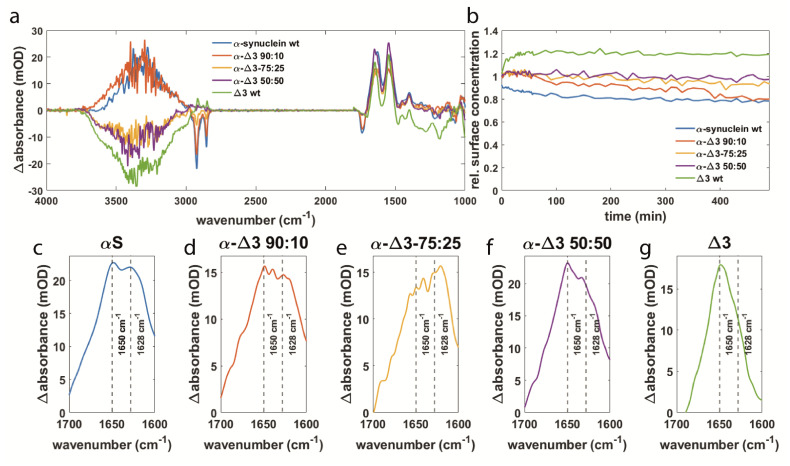
(**a**) Difference spectra of αS and Δ3 as well as mixtures of both variants at ratios of 90:10, 75:25, and 50:50 after 8 h of interaction with an SSLB. (**b**) The surface concentration of lipids over time for the same protein variants and corresponding mixtures as in (**a**). (**c**–**g**) Magnification of the amide I band from the spectra in (**a**) with decreasing αS and increasing Δ3 amounts.

The spectra of Δ3 and mixtures of Δ3 and αS are shown after 8 h of interaction with the SSLB (Figure 4a) and show similarities to those of βS (Figure 3a). This led to the hypothesis that Δ3 might interact with αS in a similar manner to βS. For a mixture of 50:50, this seems to be true since no negative lipid bands are visible, and the spectrum shows a negative water signal. However, in contrast to a 50:50 mixture of αS and βS, a component at 1628 cm^−1^ is observed for a 50:50 mixture of αS and Δ3. An explanation could be that Δ3 fulfills a similar role to βS but is less effective in doing so. These experiments have also been carried out at ratios of 75:25 and 90:10. Already at 75:25, negative lipid bands are visible (Figure 4a). At 90:10, the spectrum is almost like the one of pure αS. This behavior is also reflected in the surface concentration of the lipids on the IRE (Figure 4b). For pure Δ3, an increase is observed just like for βS. This again indicates that more lipid molecules are detected close to the IRE surface, which could be caused by the binding of lipid molecules to the C-terminus of Δ3 as postulated above for βS. By increasing the proportion of αS, a gradual decrease of the lipid surface concentration can be observed. This is similar to βS pointing towards a similar function of Δ3 as it is able to reduce the damaging of the membrane when present in comparable proportions to αS. Four of the five most common disease mutants in PD are located in exon3, indicating a prominent role of this sequence part in the pathology of the disease [56]. Furthermore, exon3 is part of the membrane binding domain of αS [17]. The important role of exon3 in αS aggregation is supported by our experiments. When Δ3 with missing exon3 is present at a comparable ratio to αS, aggregation of αS is reduced. A possible explanation could be that exon3 is important for protein–protein interactions and thus enhances aggregate formation.

The fact that we can observe similar effects for Δ3 as for βS seems to confirm the hypothesis that Δ3 might fulfill a similar physiological function in preventing αS aggregation and reducing membrane damage, although less effective than βS.

### 3.4. Orientation of Secondary Structure Elements upon Membrane Interaction Studied by Polarized Measurements

To further investigate the membrane interaction of different synuclein variants, the orientation of secondary structure elements upon membrane interaction was monitored using polarized ATR-FTIR measurements. The aim of these experiments was to analyze if differences in the orientation of secondary structure elements with respect to the membrane point towards differences in the membrane interaction of the various synuclein variants. The possibility of oligomers forming pore like structures in the membrane was reported in other studies [75,76,77]. Thus, it was of particular interest if an insertion of α-helices or aggregate structures into the membrane can be observed. Differences in the orientation of secondary structure elements could be indicative of an altered membrane interaction of different variants, which could explain the differences when it comes to membrane damage. If there are no differences in the orientation between these variants, it seems more likely that the different effects of membrane integrity and the protective function of βS and Δ3 are due to protein–protein interactions altering the aggregation behavior. An isotropic orientation of β-sheet structures could be an indicator that aggregate structures diffuse into the supernatant and are no longer in contact with the membrane.

**Figure 5:**
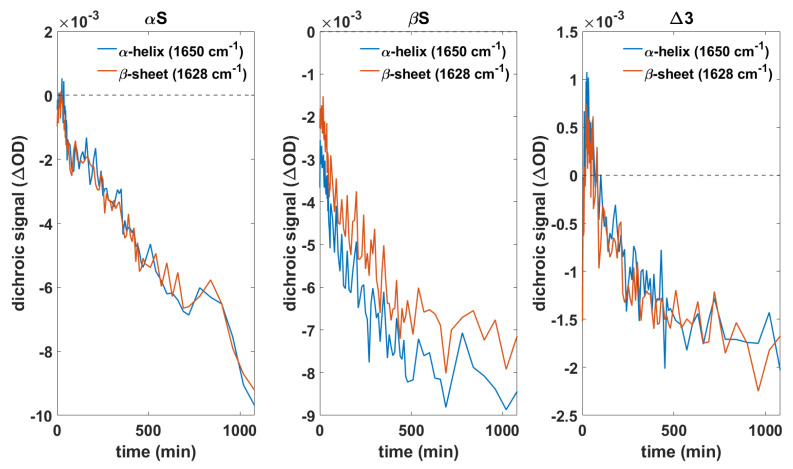
Time-dependent dichroic signals at single wavenumbers representing the orientation of α-helices (1650 cm^−1^) and β-sheet rich aggregates (1628 cm^−1^) shown for αS, βS, and Δ3.

Figure 5 shows the time-dependent dichroic signal of αS, βS, and Δ3 at 1650 cm^−1^ and 1628 cm^−1^ corresponding to α-helices and β-sheets. For all three variants, we observe negative values decreasing over time. This means that over time there is an increasing number of α-helices oriented perpendicular to the surface normal, presumably lying on top of the membrane. This is in line with previous studies describing the adoption of a membrane bound α-helical structure [16,17,18,19,20]. However, the insertion of α-helices into the membrane could not be observed. Furthermore, the β-sheet rich aggregates adopt the same orientation and form on top of the SSLB. We did not observe protein insertion into the membrane nor membrane pore formation during the studied time course. Membrane insertion and pore formation might be hindered by the solid support of the lipid bilayer or may occur on longer timescales.

### 3.5. βS and Δ3 Prevent Membrane Damage by Counteracting αS Aggregation

**Figure 6:**
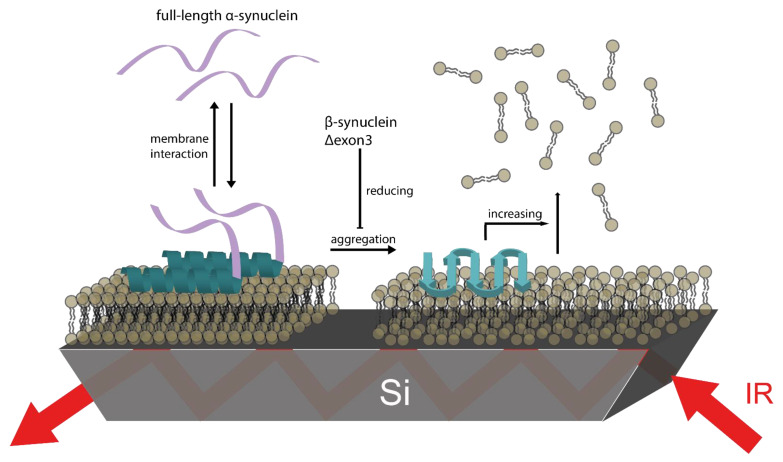
Schematic representation of the impact of βS resp. Δ3 interaction on αS aggregation. A stable SSLB is formed as biomimetic membrane on the IRE of the ATR cell. The intrinsically disordered αS binds to the membrane and forms partial α-helical structure at the N-terminus while the C-terminus remains disordered. Over time, β-sheet rich aggregates form and cause membrane disruption and the release of lipid molecules from the bilayer. The more aggregates form, the more membrane damage that occurs. The β-sheets are oriented parallel to the membrane surface. We could not observe any pore formation or insertion of the aggregates into the membrane, maybe because of the constraints of the solid support. Interaction with βS or Δ3 reduces αS aggregation and membrane damage. The effect of aggregation prevention is higher for βS than for Δ3.

Our study shows that the aggregation behavior and the effect on membrane integrity is significantly different for αS compared to βS and Δ3. All variants bind to the membrane while adopting a partial α-helical structure. However, aggregation into β-structures is only observed for αS, but not for βS and only slightly for Δ3. Membrane damage is only detected upon αS aggregation. The presence of βS or Δ3 can reduce αS aggregation and prevent damaging of the membrane. The orientation of secondary structure elements upon membrane interaction revealed no major differences between the investigated synucleins. This indicates that the differences in aggregate formation and membrane damage are due to the interaction between the synuclein variants rather than a different interaction with the membrane. Figure 6 summarizes the results schematically. Upon interaction with the SSLB, αS forms partial α-helices on top of the membrane. Aggregates form over time, thereby perturbing the membrane integrity and resulting in the release of lipid molecules from the bilayer. When αS is mixed with βS or Δ3, aggregation is reduced and thus the removal of lipids from the bilayer is decreased. An increase in the lipid surface concentration has been observed for βS and Δ3, and mixtures of αS with a sufficient fraction of βS or Δ3. This indicates that lipids from the supernatant might bind to the C-terminus of αS or the other synuclein variants.

The orientation of the secondary structure elements with respect to the surface of the SSLB was roughly the same for all variants. As illustrated in Figure 6, α-helices and β-sheets were oriented parallel to the surface and seemed to simply lie on top of the membrane. This indicates that a similar protein–membrane interaction for the different variants follows a similar mechanism. Therefore, the differences in aggregation and membrane damage are rather linked to differences in the sequence of these variants. Δ3 lacks exon3 completely and βS differs from αS at three sites within this exon emphasizing the crucial role of exon3. Since our results suggest a similar protein–membrane interaction for the synuclein variants, it could be surmised that exon3 has an important role in protein–protein interaction. The ability of αS to form aggregates with synuclein variants without an identical exon3 appears to be reduced significantly. It is reported that temperature has an effect on synuclein-membrane interaction [78,79] and we expect an influence on binding affinity and aggregation kinetics detectable in future temperature-dependent ATR-FTIR studies.

## 4. Conclusions

Our ATR-FTIR study reveals that βS and Δ3 reduce αS aggregation and membrane damage when present in comparable proportions to αS. Since there was no difference in the orientation of the secondary structure elements upon membrane interaction, it seems likely that βS and Δ3 show different protein–protein interaction with αS rather than altering the interaction with the membrane. Our results confirm the protective role of βS when it comes to αS aggregation. Additionally, we could show that Δ3 can exhibit a similar function to βS, preventing aggregation of αS and membrane damage, although less effective than βS. Altered expression levels of αS and βS have been linked to PD [47]. Diminished levels of Δexon3 have been reported for patients suffering from DLB and AD and increased levels of Δexon3 in PD [60]. Our findings demonstrate how crucial the ratio between these variants is to prevent membrane damage and support the assumption that a misbalance between synuclein variants could contribute to disease progression of PD and other related synucleinopathies.

## Supplementary Materials

Supplementary information for this paper is available online at https://www.mdpi.com/article/10.3390/biom11081067/s1. Figure S1: Silver stained polyacryl gel, Figure S2: Coomassie stained polyacrylamide gel, Figure S3: Elution profile, Figure S4: Control measurements with diluted α-synuclein, Figure S5: Control measurements with PLL.
