# Supplementary material for: Synuclein Family Members Prevent Membrane Damage by Counteracting α-Synuclein Aggregation
Source: Biomolecules. 2021 Jul 21;11(8):1067. doi: 10.3390/biom11081067 (PMC8392020; doi:10.3390/biom11081067)
Supplement: Supplementary file 1 [file biomolecules-11-01067-s001.zip › biomolecules-1277172-supplementary.pdf]

Supporting Information

Synuclein family members prevent membrane damage by counteracting α-synuclein aggregation

Christian Scheibe,[1] Christiaan Karreman,[2] Stefan Schildknecht,[2,3] Marcel Leist, [2]

Karin Hauser[1]*

[1] Department of Chemistry, University of Konstanz, 78457 Konstanz, Germany

[2] Department of Biology, University of Konstanz, 78457 Konstanz, Germany

[3]Department of Life Sciences, Albstadt-Sigmaringen University of Applied Sciences, 72488 Sigmaringen, Germany

* Correspondence: karin.hauser@uni-konstanz.de

Protein Purification

Isolation of α-synuclein and its variants

Recombinant proteins were produced in *E. coli* (Tuner(DE3)) and subsequently purified by a three steps procedure. The first step was the boiling of the resuspended bacterial cells in a small volume of PBS followed by centrifugation at 14,800 g for 20 minutes. This step removed a lot of bacterial proteins (see Figure S1, lane 2 compared to lane 3). The second step that is responsible for removal of most other proteins is the use of a Capture-select column (antibody based affinity chromatography) (see lane 5). The third step that removed the remainder of the contaminating proteins was an acetone precipitation. The latter was included as some preparations contained some lipophilic bacterial protein even after the Capture-select column. See Figure S2.

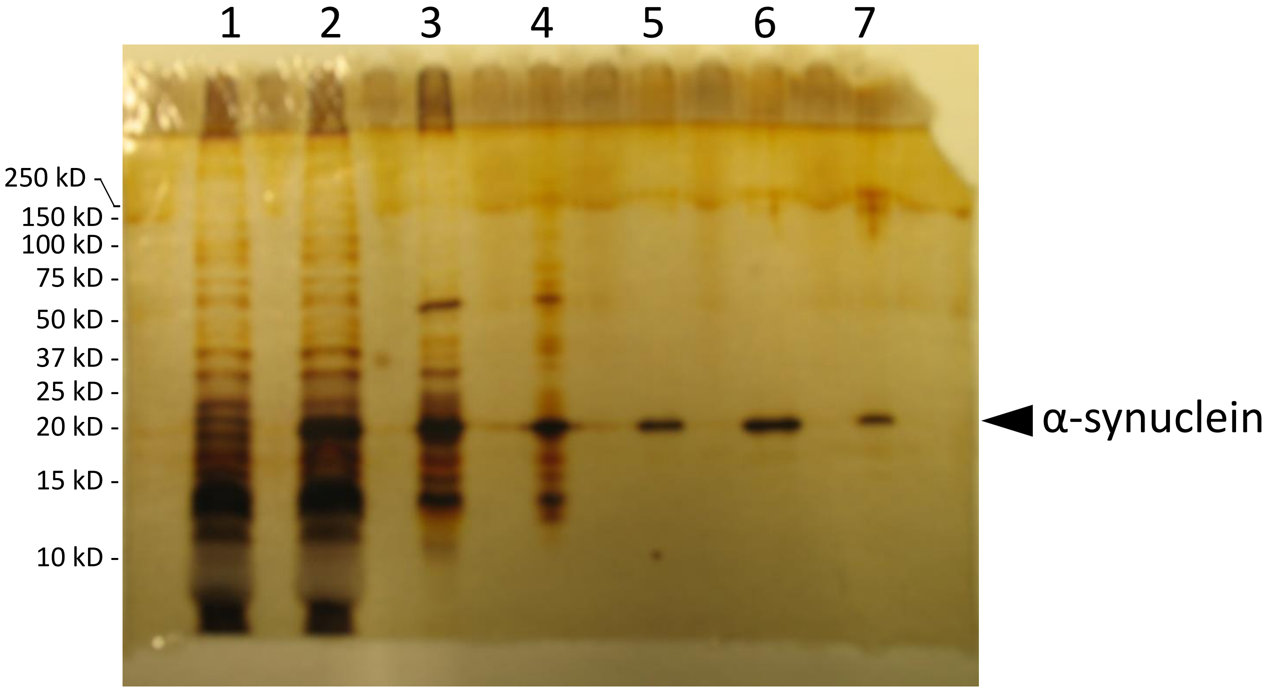

Figure S1. Silverstained polyacryl gel showing the purity of α-synuclein after each step in the standard protocol.

The numbered lanes were loaded as follows:

1) Lysate of uninduced bacteria, 2) Lysate of induced bacteria, 3) Lysate after boiling, 4) Pellet after $(NH_4)_2SO_4$, 5) After nanobody-Column, 6) After de-salting, 7) Concentrated (acetone precipitation)

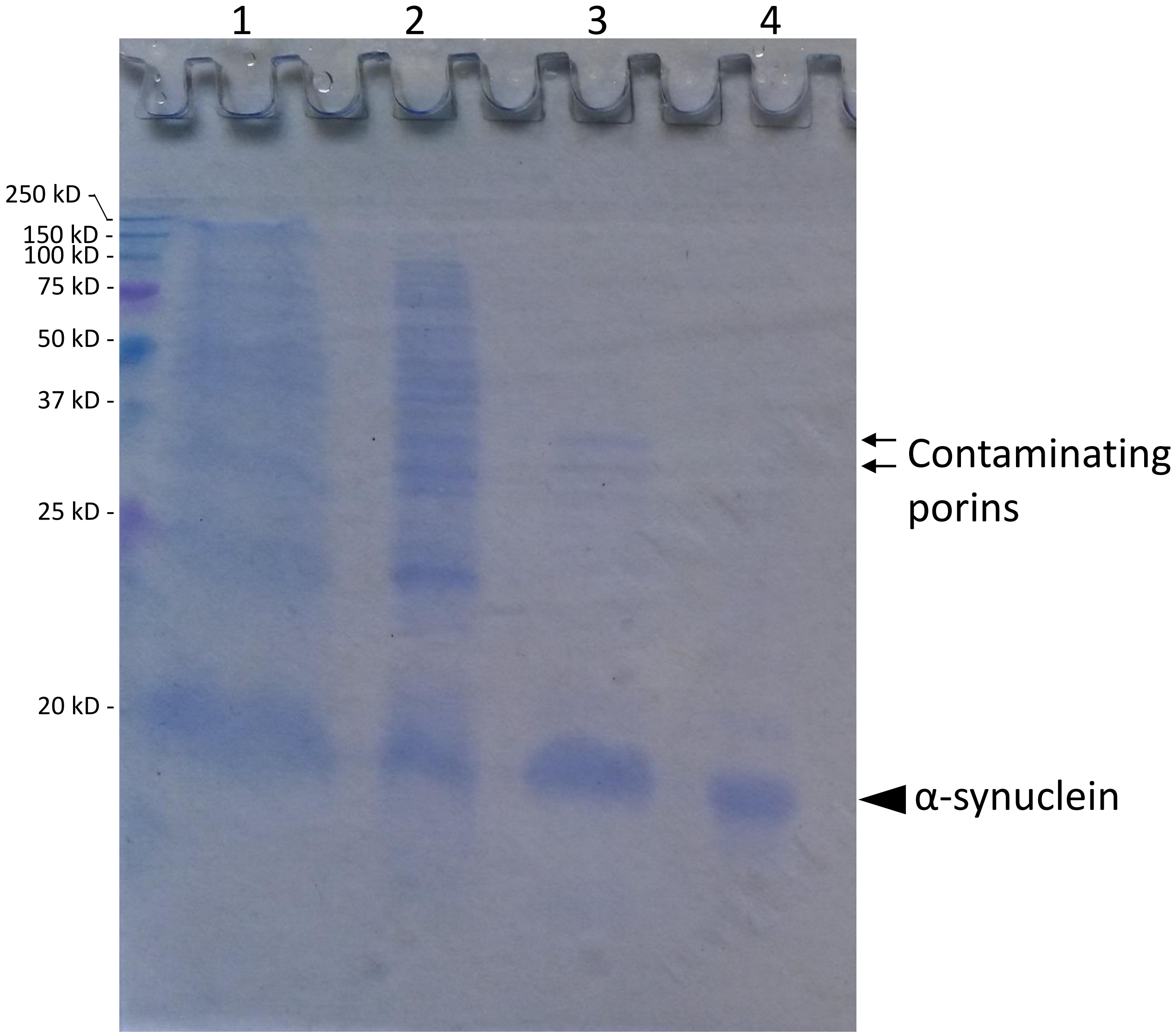

Figure S2 Picture of Coomassie stained polyacrylamide gel gel showing the purity of α-synuclein in four crucial steps.

The numbered lanes were loaded as follows: 1) Induced lysate, 2) Lysate after boiling and centrifugation, 3) After Capture-select column and de-salt, 4) Concentrated (acetone). Note that after the Capture-select immunoaffinity column there is still a contamination in lane 3 (arrows).The bands were identified by MS as the porin proteins A and F (mpA & ompF)

For the purification of α-synuclein via the Capture-select beads a 25 ml column was used connected to an FPLC system. For a typical elution profile see Figure S3. The salt of the positive fractions was removed by applying the pooled fractions to a HiPrep 26/10 desalting column of GE Healthcare (now Cytiva). The column was equilibrated with pure water.

α-synuclein and its variants were precipitated by adding 4 volumes of precooled acetone (-20 °C) and centrifuged. The pellet was dried under vacuum and weighted. These pellets were stored at -20 °C.

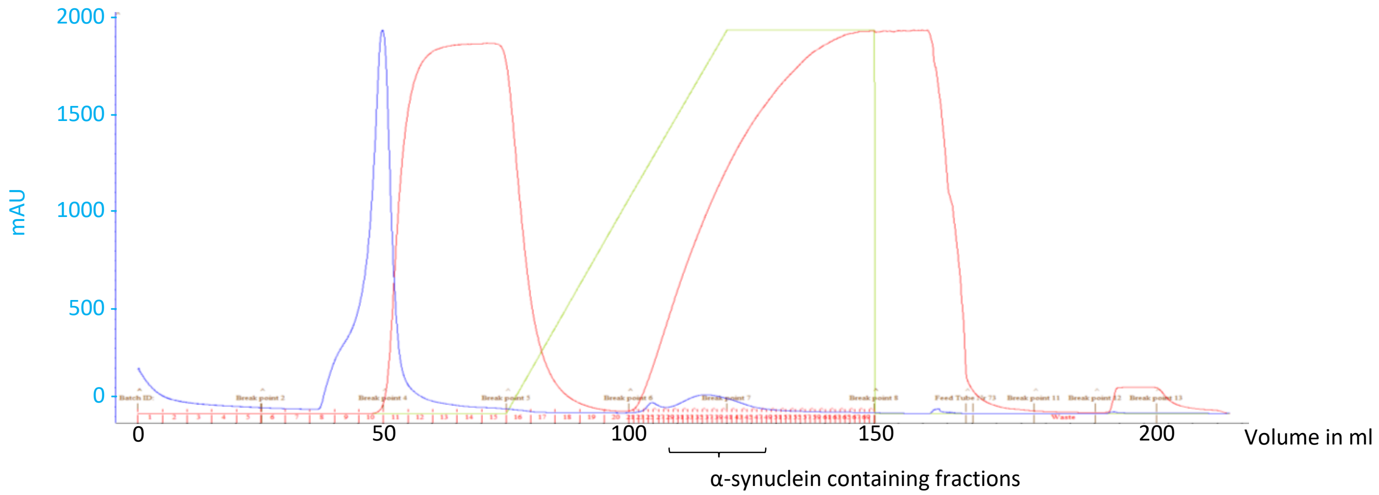

Figure S3. Typical elution profile of α-synuclein on Capture-select beads filled column for FPLC. In blue the absorption at 280 nm, in red conductivity and in green the percentage of the programmed second buffer. The numbers of the collected fractions are given at the bottom. The loading of the crude bacterial lysate is not shown, the absorption of the flow-through would dwarf all other peaks.

The loaded column was washed with 25 ml (1 x the volume of the column) 10 mM Tris pH 7.4, followed by a wash of 25 ml of 10 mM Tris pH 7.4 with 2 M NaCl. All unspecifically bound proteins (either bound to the beads or to α-synuclein) were washed off, as shown by the peak at fraction 10/11. They come at the very front of the salt peak that stretches from fraction 11-17.

This was followed by another wash with 25 ml 10 mM Tris pH 7.4.

The elution of α-synuclein is accomplished by applying a gradient up to 2 M $MgCl_2$. The positive fractions that peak at fraction 36, contain approx. 1 M $MgCl_2$.

ATR-FTIR Spectroscopy

Penetration Depth of the Evanescent Field

In ATR-FTIR spectroscopy infrared light is directed into an internal reflection element (IRE). This is typically a trapezoidal crystal that is transparent for infrared light. The IRE material has a refractive index higher than that of the surrounding sample medium. The incident light is totally reflected several times at the upper and lower IRE surfaces. At each reflection point, an evanescent wave penetrates the sample medium with lower refractive index, probes the molecules close to the IRE surface and can thus be used for spectroscopic measurements [1].

The strength of the evanescent field decays exponentially with the distance to the interface of the IRE z and is described by

$$E(z) = E_0 e^{-z/d_p}$$

where E(z) is the strength of the electric field, E_0 is the strength of the electric field at the interface and d_p the penetration depth. The penetration depth d_p can be determined using

$$d_p = \frac{\lambda}{n_1 2\pi(\sin^2\theta - n_{21}^2)^{1/2}}$$

where λ is the wavelength, θ the incidence angle, n_1 the refractive index of the IRE and n_{21} = n_2/n_1 with n_2 being the refractive index of the medium on top of the IRE. Larger wavelengths or smaller angles of incidence increase the penetration depth. The angle of incidence is limited by the critical angle for total reflection. For ATR-FTIR spectroscopy the penetration depth is typically in the µm range [1].

When using polarized ATR-FTIR spectroscopy, the penetration depth depends on the polarization of the incidence light. The penetration depth for perpendicular polarized light is determined via

$$d_{p\perp} = \frac{n_{21}\cos\theta}{\pi(1-n_{21}^2)(\sin^2\theta - n_{21}^2)^{1/2}} \cdot \lambda_1$$

and for parallel polarized light via

$$d_{p\parallel} = \frac{n_{21}\cos\theta(2\sin^2\theta - n_{21}^2)}{\pi(1-n_{21}^2)[(1+n_{21}^2)\sin^2\theta - n_{21}^2](\sin^2\theta - n_{21}^2)^{1/2}} \cdot \lambda_1,$$

with λ_1 being λ/n_1 [1]. For an IRE made of silicon with n_1 = 3.42, n_2 = 1.55 [1] and an incidence angle of 45° the penetration depth at 1650 cm^{-1} is $d_{p\perp} = 0.42\ \mu m$ for perpendicular polarized light and $d_{p\parallel} = 0.84\ \mu m$ for parallel polarized light [1].

Using Dichroic Spectra to investigate Molecular Orientation

The orientation of molecules can be analyzed with polarized ATR-FTIR spectroscopy and dichroic spectra. The absorption of polarized infrared light depends on the orientation of the transition dipole moment compared to the electric field vector of the incidence light. Dichroic spectra were obtained by subtracting the weighted spectrum recorded with perpendicular polarized light from the spectrum recorded with parallel polarized light. R_{iso} was used as weighting factor and is defined below. Without any preferred orientation the subtraction should

yield a straight horizontal line. A positive signal indicates that the transition dipole moment is oriented parallel to the surface normal. A negative signal on the other hand indicates an orientation of the transition dipole moment that is perpendicular to the surface normal [1].

Dichroic spectra were used to determine the orientation of secondary structure elements upon interaction with a SSLB. The difference spectra were calculated by using MatLab (Mathworks, USA) and a weighting factor R_{iso}.

Determination of the Dichroic Ratio R_{iso}

To compensate for the different penetration depths depending on the polarization a weighting factor R_{iso} for the calculation of the dichroic spectra is required. The absorption band of an isotropically oriented molecular group can be used to determine R_{iso}. This is done by calculating the ratio of the integrated absorbances from the spectra recorded with parallel ($A_\parallel$) and perpendicular ($A\perp$) polarized light according to $R_{iso} = A_\parallel / A_\perp$. This was done using the lipid carbonyl band at 1740 cm^{-1} which has also been used in other studies [2].

Control Measurements

Membrane Damage of Diluted α-Synuclein

Our data shows that the presence of other synuclein variants can reduce αS aggregation. However, the concentration of αS is lower when mixed with βS and Δ3 compared to the experiments with pure αS. Previous studies have reported that the aggregation of αS is increased at higher protein concentrations and decreased at lower protein concentrations [3]. To ensure that the reported results are due to the presence of the other synuclein variants and not due to lowered αS concentrations, control measurements have been performed where αS was diluted to half of the concentration (0.5 mg/ml) compared to the previous experiments with αS (1 mg/ml).

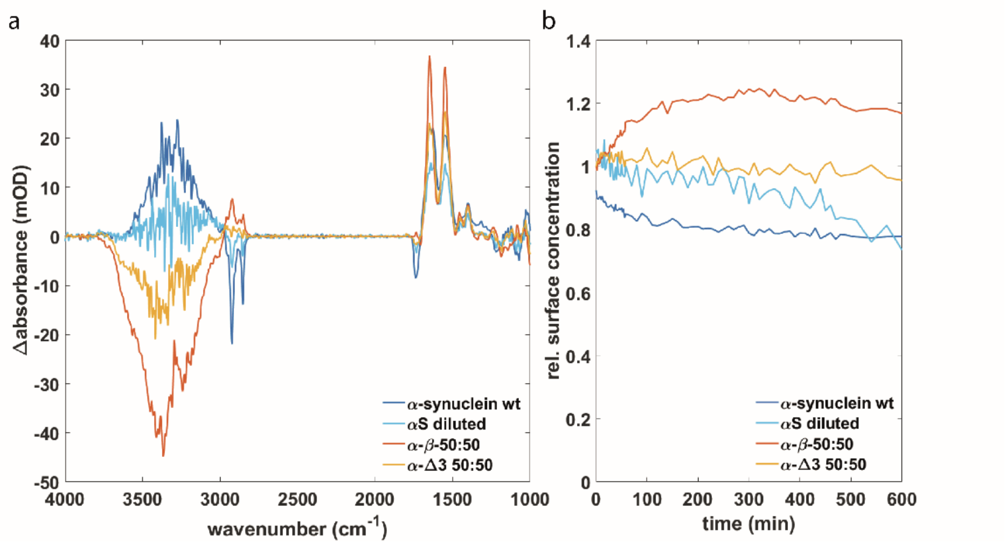

Figure S4. (a) Difference spectra of pure αS and 50:50 mixtures of αS with buffer solution, βS and Δ3. (b) relative surface concentration of the lipids upon interaction with the samples from (a).

The data in Fig. S4 shows the control experiment with diluted αS in comparison to αS at higher concentration and the 50:50 mixtures with βS and Δ3, all already presented in Figures 3 and 4 in the main text. The signs of the absorption bands for diluted αS are same as for αS at higher concentrations and opposite to those for mixtures with βS (Fig. S4a). The surface concentration of the lipids (Fig. S4b) is decreasing upon interaction with lower concentrated αS. This process is slower compared to the higher αS concentration, but after 500-600 minutes the relative surface concentration of the lipids at both αS concentrations is comparable. In the later stages of the experiment, the surface concentration of the lipids upon interaction with lower concentrated αS is lower as than the one upon interaction with the 50:50 mixtures of αS with βS and Δ3. This indicates that membrane damage is not prevented by diluting αS. Therefore, the prevention of membrane damage must be due to the interaction between the different synuclein variants.

Orientation of Poly-L-Lysine

Poly-L-Lysine (PLL) has been reported to undergo a transition from random coil to an α-helical structure upon interaction with negatively charged phospholipid vesicles at neutral pH [4]. Because of this similarity to αS, PLL was chosen to compare the time-dependent dichroic signal of PLL with the one for the different synuclein variants.

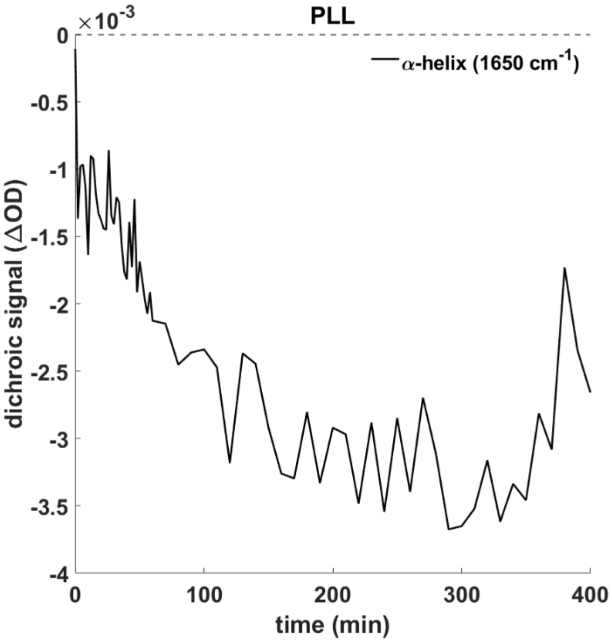

Figure S5. Dichroic signal for α-helices formed upon membrane interaction of PLL.

For PLL the time course of the dichroic signal (Fig. S5) is similar to the different synuclein variants (Fig. 5, main text). Upon membrane interaction the formation of α-helices is observed, and the dichroic signal is negative and decreasing with time indicating that an increasing number of α-helices is orienting parallel to the surface over time. Since there is no membrane damage observed for PLL interacting with the membrane it can be assumed that the helices are simply lying on top of the membrane. This shows that the formation of an α-helix on a lipid membrane does not necessarily cause membrane damage. Thus changes in lipid surface

concentration respectively membrane damage must be caused by the unique properties of synuclein variants and their interaction with the membrane and with each other.

1. Goormaghtigh, E.; Raussens, V.; Ruysschaert, J.M. Attenuated total reflection infrared spectroscopy of proteins and lipids in biological membranes. *Biochim Biophys Acta* **1999**, *1422*, 105-185.
2. Zhang, G.; Keiderling, T.A. Equilibrium and Dynamic Spectroscopic Studies of the Interaction of Monomeric β-Lactoglobulin with Lipid Vesicles at Low pH. *Biochemistry* **2014**, *53*, 3079-3087.
3. Afitska, K.; Fucikova, A.; Shvadchak, V.V.; Yushchenko, D.A. alpha-Synuclein aggregation at low concentrations. *BBA-Proteins Proteomics* **2019**, *1867*, 701-709.
4. Trombik, P.; Cieślik-Boczula, K. Influence of phenothiazine molecules on the interactions between positively charged poly-l-lysine and negatively charged DPPC/DPPG membranes. *Spectrochim Acta A: Mol Biomol Spectr* **2020**, *227*, 117563.
